# Methyldiethanolamine: MAK Value Documentation, addendum – Translation of the German version from 2024

**DOI:** 10.34865/mb10559kske11_1ad

**Published:** 2026-03-30

**Authors:** Andrea Hartwig, MAK Commission

**Affiliations:** 1 Institute of Applied Biosciences, Department of Food Chemistry and Toxicology, Karlsruhe Institute of Technology (KIT) Adenauerring 20a, Building 50.41 76131 Karlsruhe Germany; 2 Permanent Senate Commission for the Investigation of Health Hazards of Chemical Compounds in the Work Area, Deutsche Forschungsgemeinschaft, Kennedyallee 40, 53175 Bonn, Germany. Further information: Permanent Senate Commission for the Investigation of Health Hazards of Chemical Compounds in the Work Area | DFG

**Keywords:** methyldiethanolamine, liver, kidney, carcinogenicity, irritation, MAK value, maximum workplace concentration, peak limitation, skin absorption, read across, Luft, air

## Abstract

The German Senate Commission for the Investigation of Health Hazards of Chemical Compounds in the Work Area (MAK Commission) summarized and re-evaluated the data for methyldiethanolamine [105-59-9] to derive an occupational exposure limit value (maximum concentration at the workplace, MAK value) considering all toxicological end points. Relevant studies were identified from a literature search and also unpublished study reports were used. There are no studies investigating the carcinogenic effects of methyldiethanolamine. Methyldiethanolamine is a methylated metabolite of diethanolamine. Diethanolamine increased the incidence of liver and kidney tumours in a dermal carcinogenicity study in mice at the lowest dose tested of 40 mg/kg body weight and day. Mechanistic studies indicate that methyldiethanolamine, like diethanolamine, may disrupt choline homoeostasis, which is thought to have caused the liver tumours. The same mechanism has been suggested for kidney tumours, but has not been experimentally proven. Human relevance for this mechanism cannot be ruled out and methyldiethanolamine, like diethanolamine, has therefore been classified in Carcinogen Category 3. Methyldiethanolamine is neither mutagenic nor clastogenic. For this reason, despite its classification in Carcinogen Category 3, a MAK value can be derived. Studies with repeated inhalation exposure to methyldiethanolamine are not available. In a comparison of structurally related ethanolamines, methyldiethanolamine was found to lie between diethanolamine and triethanolamine in terms of irritation and basicity. Diethanolamine, which is a much stronger irritant than methyldiethanolamine, has a MAK value of 1 mg/m^3^. On this basis, a MAK value of 2 mg/m^3^ has been established for methyldiethanolamine. Aerosol impaction is not expected to occur because this concentration is below vapour saturation. The margin between the MAK value of 2 mg/m^3^ and the concentration at which a carcinogenic effect may occur is sufficiently large. Peak Limitation Category I with an excursion factor of 1 has been derived in analogy to the other ethanolamines. The only available study investigating developmental toxicity of methyldiethanolamine was carried out according to OECD Test Guideline 421. As this study does not include a full investigation of teratogenicity, methyldiethanolamine has been assigned to Pregnancy Risk Group D. Based on studies in rats, percutaneous absorption is expected to contribute significantly to systemic toxicity. Therefore, methyldiethanolamine has been designated with “H”. The limited results from animal studies do not suggest a skin-sensitizing potential. Furthermore, in spite of widespread use, only two cases involving occupational exposure have been reported. Data for respiratory sensitization are not available.

**Table:** 

**MAK value (2023)**	**2 mg/m^3^ ≙ 0.4 ml/m^3^**
**Peak limitation (2023)**	**Category I, excursion factor 1**
	
**Absorption through the skin (2023)**	**H**
**Sensitization**	**–**
**Carcinogenicity (2023)**	**Category 3**
**Prenatal toxicity (2023)**	**Pregnancy Risk Group D**
**Germ cell mutagenicity**	**–**
	
**BAT value**	**–**
	
Synonyms	*N*,*N*-bis(2-hydroxyethyl)methylamine diethanolmethylamine methylbis(2-hydroxyethyl)amine *N*-methyldiethanolamine methyliminodiethanol 2,2′-(methylimino)diethanol *N*-methyl-2,2′-iminodiethanol
Chemical name (IUPAC)	2-[2-hydroxyethyl(methyl)amino]ethanol
CAS number	105-59-9
Structural formula	H_3_C–N(CH_2_–CH_2_OH)_2_
Molecular formula	C_5_H_13_NO_2_
Molar mass	119.16 g/mol
Melting point	–21.3 °C (ECHA 2022)
Boiling point at 1013 hPa	243.3 °C (ECHA 2022)
Density at 20 °C	1.04 g/cm^3^ (ECHA 2022)
Vapour pressure	0.0031 hPa at 20 °C (ECHA 2022)
log K_OW_	–1.16 at 23 °C, pH 10.5 (OECD TG 107; ECHA 2022)
Solubility	1000 g/l water at 20 °C (ECHA 2022)
pKa value	8.52 at 25 °C; pH 11.5 for 10% solution (ECHA 2022)
**1 ml/m^3^ (ppm) ≙ 4.944 mg/m^3^**	**1 mg/m^3^ ≙ 0.202 ml/m^3^ (ppm)**
	
Hydrolytic stability	hydrolysis is not expected (ECHA 2022)
Stability	no data
Production	from methylamine and ethylene oxide as a mixture of methylmonoethanolamine and methyldiethanolamine (Greim 1998)
Purity	at least 98% (Greim 1998)
Impurities	no data
Uses	component of metal-working fluids, intermediate for the chemical industry, corrosion inhibitor (Greim 1998), in coatings and paints, for the manufacture of foams and elastomers, as a pH buffer and as an anti-corrosion additive in metal-working fluids; methyldiethanolamine forms quat salts with fatty acids, which are then used in fabric softener formulations (BASF SE 2022); for the separation of CO_2_, H_2_S and other acidic gases from natural gas (SensoTech GmbH 2016)
Bans on usage	methyldiethanolamine is subject to the Chemical Weapons Convention and is listed there as a precursor (OPCW 2022)
Concentrations used	maximum of 10% in metal-working concentrates (Hartwig and MAK Commission 2023, available in German only)

Note: forms nitrosamines; the substance can occur simultaneously as vapour and aerosol.

Documentation for methyldiethanolamine was published in 1993 (Greim 1998); no MAK value was established at that time because the database was inadequate. New data have become available and this addendum reviews whether a MAK value can be derived. In addition, the end points prenatal toxicity, genotoxicity, carcinogenicity, sensitization and absorption through the skin have been re-evaluated. Unpublished toxicological studies from companies were made available to the Commission.

## 1 Toxic Effects and Mode of Action

Methyldiethanolamine causes only low acute toxicity. The urine is the primary route of excretion after absorption through the skin with an elimination half-life of more than 30 hours.

Methyldiethanolamine is only mildly irritating to the skin of rabbits after a single application, but is irritating to the eyes of rabbits.

No studies are available for the effects induced by repeated oral or inhalation exposure. In a dermal study in F344 rats with exposure for 11 days, undiluted methyldiethanolamine led to marked skin irritation, haematological and clinico-chemical changes and reduced body weight gains at the low dose of 260 mg/kg body weight and day and above. When a 50% aqueous solution was applied, these findings were observed only at doses of 500 mg/kg body weight and day and above. After dermal application for 13 weeks, slight, transient decreases in body weight gains were observed in the males at 100 mg/kg body weight and day (10% aqueous solution) and above; this effect was not considered adverse. This finding was more pronounced at 250 mg/kg body weight and day and above (25% solution) and irritation developed.

Animal studies did not yield evidence of skin-sensitizing potential. Only two cases involving occupational exposure have been reported despite the widespread use of methyldiethanolamine.

In a screening test for reproductive toxicity and developmental toxicity carried out in male and female Wistar rats according to OECD Test Guideline 421, a longer gestation period, litter loss, a reduced survival index, reduced body weight gains in the offspring and impaired lactation behaviour were observed at the highest gavage dose of 1000 mg/kg body weight and day. Initial effects on body weights and body weight gains were observed in the parental animals at 300 mg/kg body weight and day and above.

Methyldiethanolamine did not have genotoxic effects in vitro in bacteria, in HPRT or SCE tests in mammalian cells or in the only genotoxicity test that was carried out in vivo, a mouse micronucleus test.

There are no studies of carcinogenic effects available. Methyldiethanolamine is the methylated metabolite of diethanolamine. In a dermal carcinogenicity study, diethanolamine increased the incidence of liver and kidney tumours in B6C3F1 mice at dose levels of 40 mg/kg body weight and day and above. The mechanistic studies showed that methyldiethanolamine, like diethanolamine, may disrupt choline homoeostasis, which is considered the cause of the liver tumours. The same mechanism has been suggested for the kidney tumours, but this has not been substantiated by the experimental findings. Therefore, human relevance cannot be ruled out completely.

## 2 Mechanism of Action

### 2.1 Irritation of the skin

According to the authors of the study with repeated dermal application in F344 rats (see Section 5.2.3), the skin irritation caused by methyldiethanolamine is due to its hygroscopic effects (no author 2022) and its persistence in the skin. Percutaneous pharmacokinetic studies demonstrated that the substance is first absorbed into the skin and then released slowly into the circulating blood (Werley et al. 1997). Also, skin irritation may be caused by the alkalinity of the substance.

### 2.2 Carcinogenicity

In a dermal carcinogenicity study carried out by the NTP, the structurally related diethanolamine increased the incidence of liver and kidney tumours in B6C3F1 mice at the lowest dose tested of 40 mg/kg body weight and day and above. The tumour incidences in F344 rats were not increased (Hartwig 2015 b).

**Fig. 1:**
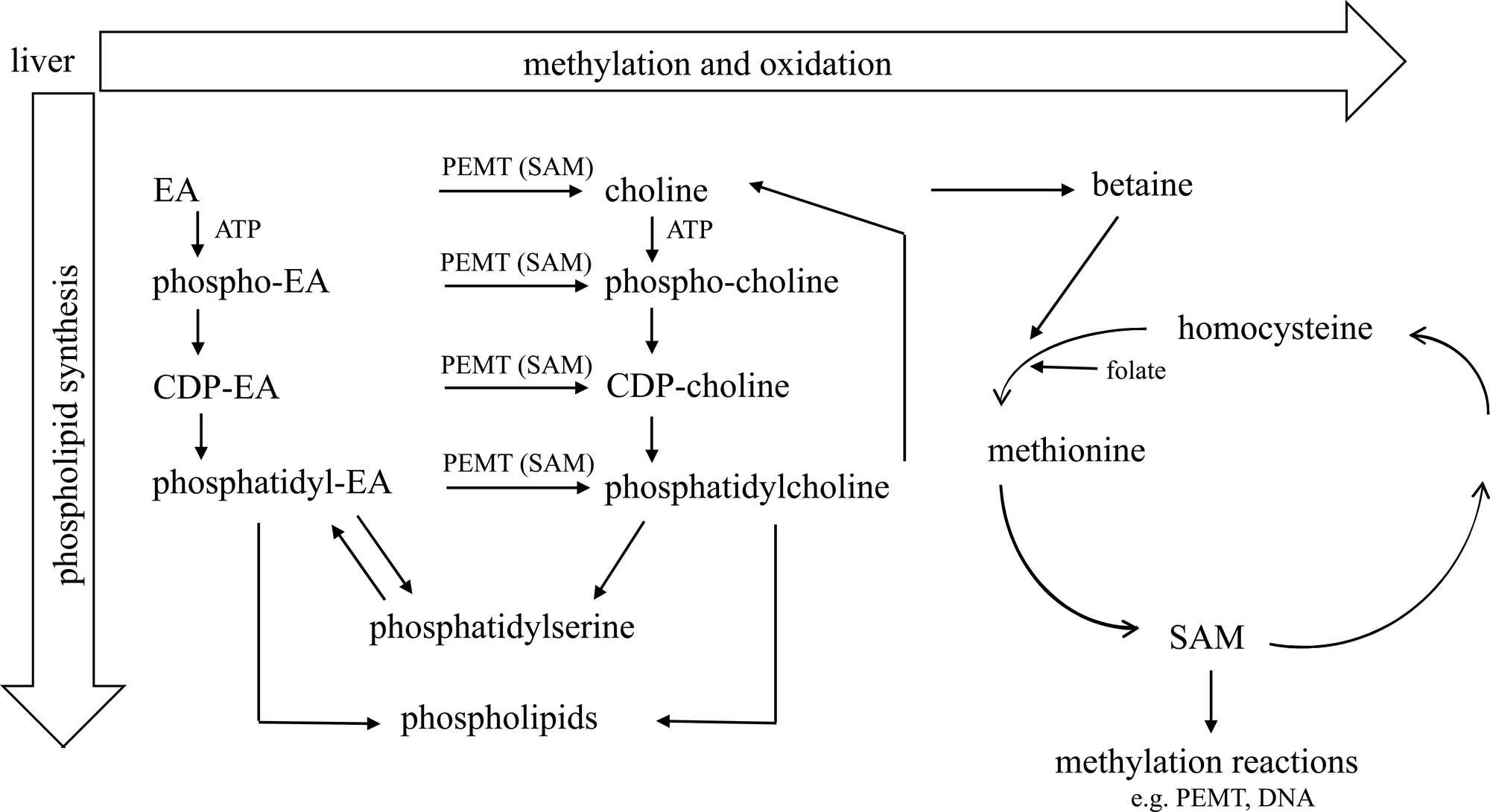
Choline metabolism in the liver (according to Kirman et al. 2016)

ATP: adenosine triphosphate; CDP: cytidine diphosphate; EA: ethanolamine; PEMT: phosphatidylethanolamine *N*-methyltransferase; SAM: *S*-adenosyl methionine; SAH: *S*-adenosyl homocysteine

The main mechanism proposed for the carcinogenic effects induced by diethanolamine is the disruption of choline homoeostasis. Methyldiethanolamine is a metabolic product that forms after *N*-methylation. Choline is necessary for the synthesis of phospholipids (phosphatidylcholine, phosphatidylethanolamine and sphingomyelin; produced in all tissues), betaine (mainly in the liver and kidneys) and acetylcholine (mainly in nerve tissue). Choline is taken up with the diet or recycled from phospholipids. Choline may be synthetized from ethanolamine by methylation (using *S*-adenosyl methionine (SAM) as a methyl donor) and then oxidized to betaine (see Figure 1). Ethanolamine and choline may undergo further phosphorylation and metabolism and then be incorporated into phospholipids. Betaine serves as a methyl donor for homocysteine and thus contributes to the formation of SAM, which plays an important role in methylation reactions in the cell. The liver requires more choline than other tissues because it synthesizes phospholipids, which are secreted into the plasma and intestinal lumen. The kidneys likewise require more choline because betaine is an important osmolyte (Kirman et al. 2016).

The mechanistic studies that were carried out mainly in mice as a result of the tumour findings demonstrated both in vitro and in vivo that diethanolamine disrupts choline homoeostasis, thereby increasing cell proliferation. The data are described in detail in the review published by Kirman et al. (2016) and are summarized below.

**Fig. 2:**
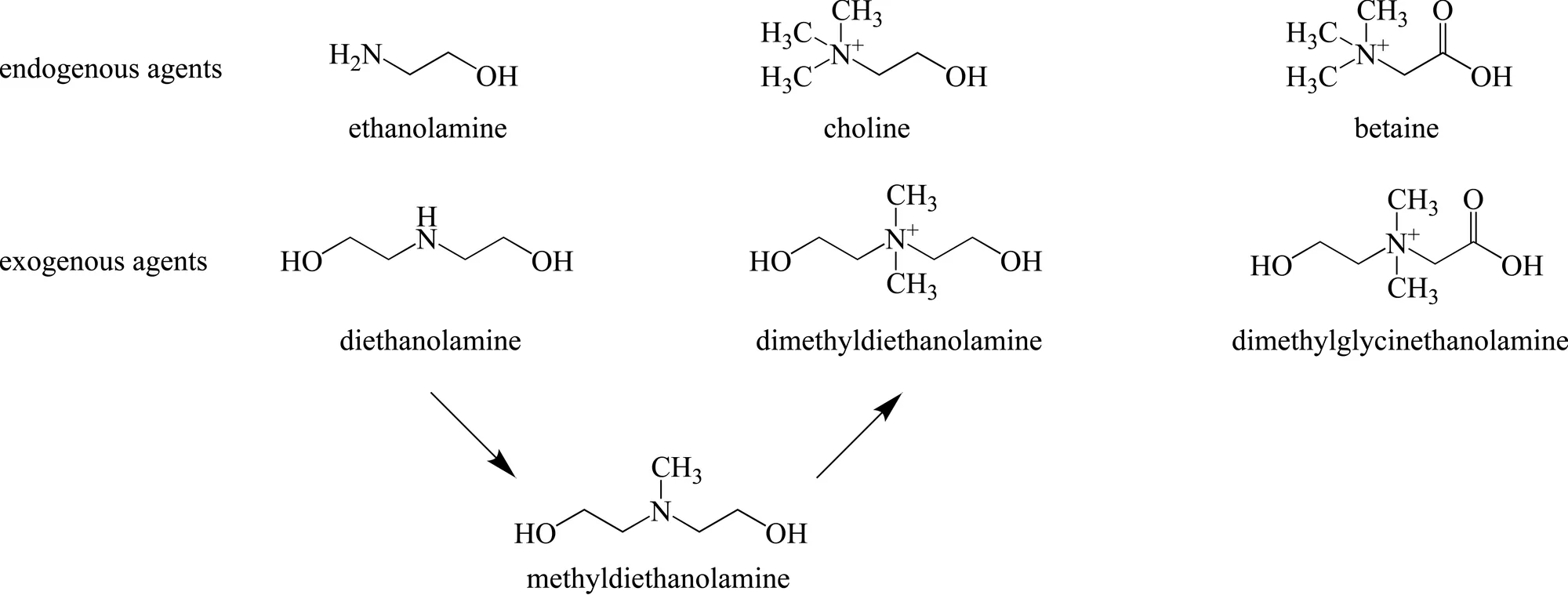
Comparison of the structure of diethanolamine and its metabolites with that of the endogenous analogues of choline metabolism (according to Kirman et al. 2016)

Diethanolamine and its methylated metabolites are structural analogues of endogenous substances that are important for choline homoeostasis (see Figure 2). Adequate data are available to substantiate an epigenetic mechanism in the formation of liver tumours in mice that involves a disturbance in choline homoeostasis and hepatic methylation reactions. This mechanism of action presumably applies also to kidney tumours; however, there is no direct experimental evidence of key events in the kidneys (Kirman et al. 2016).

**Fig. 3:**
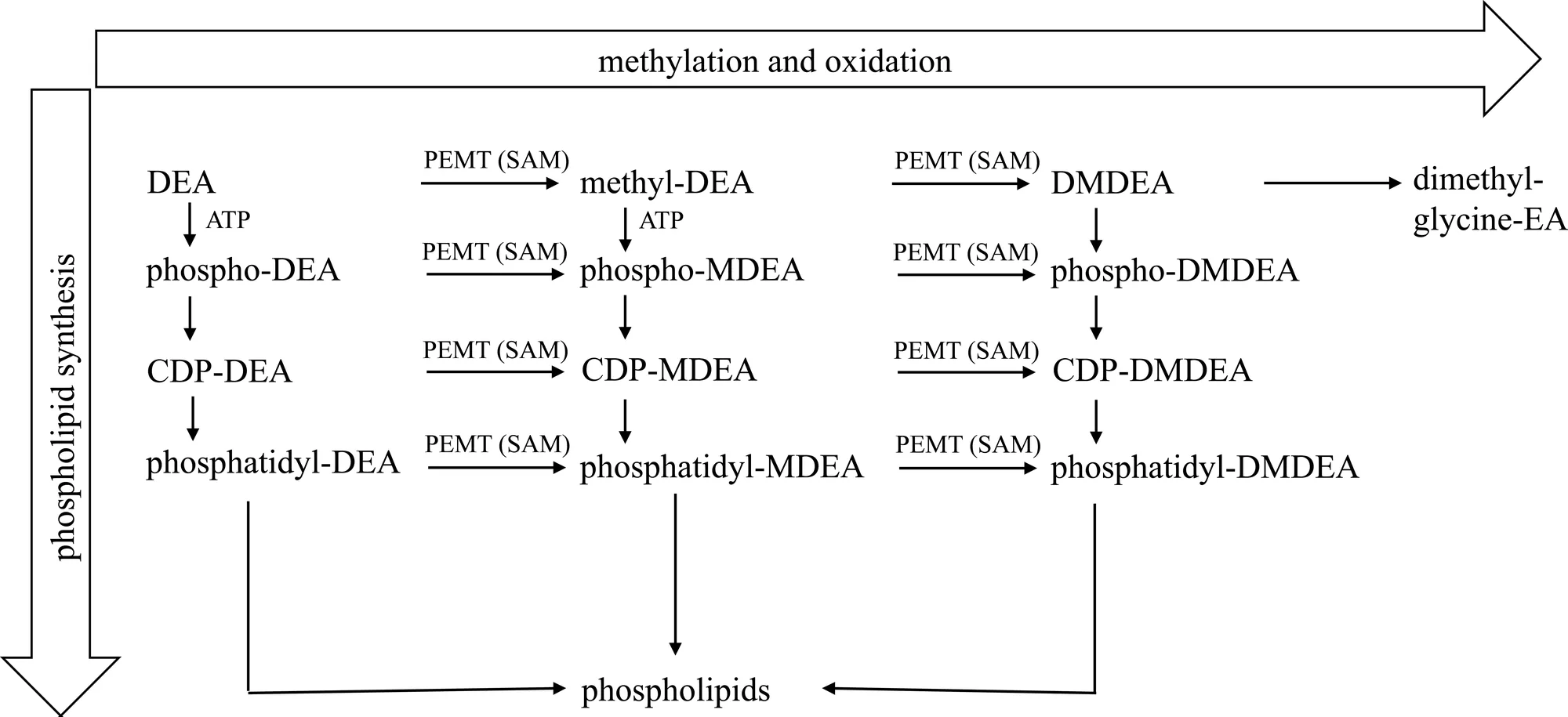
Incorporation of diethanolamine and methyldiethanolamine into the endogenous phospholipid and choline metabolism (according to Kirman et al. 2016)

ATP: adenosine triphosphate; CDP: cytidine diphosphate; DAG: diacylglycerol; DEA: diethanolamine; DMDEA: dimethyldiethanolamine; EA: ethanolamine; MDEA: methyldiethanolamine; PEMT: phosphatidylethanolamine *N*-methyltransferase; SAM: *S*-adenosyl methionine

Studies in rats have shown that the metabolism of diethanolamine follows two general pathways (see  Figure 3). The first involves the methylation of diethanolamine via *S*-adenosyl methionine (SAM), and the second involves phosphorylation of the substance followed by its incorporation into phospholipids. Methyldiethanolamine is the first methylated metabolite; it may also be incorporated into phospholipid metabolism. Methyldiethanolamine is either further methylated to form dimethyldiethanolamine, which is oxidized to dimethylglycinethanolamine, or phosphorylated to form phosphatidylmethyldiethanolamine (Kirman et al. 2016).

The metabolic pathways followed by diethanolamine and methyldiethanolamine are very similar to that of choline; it is assumed that there is overlapping of the same enzyme systems. Dimethyldiethanolamine, which forms from methyldiethanolamine, is a structural analogue of choline. Diethanolamine is a structural analogue of ethanolamine, and dimethylglycine is a structural analogue of betaine. As a result of the extensive overlap in metabolic pathways, diethanolamine and its metabolites can perturb choline homoeostasis at multiple points. They mainly involve the inhibition of choline transport, a decreased SAM concentration, decreased phospholipid synthesis and a reduced betaine concentration (Kirman et al. 2016).

Diethanolamine and methyldiethanolamine may be involved in the reduction of the hepatic SAM concentration in at least two ways: (1) a decrease in the choline and betaine concentrations, which results in the availability of less betaine for the donation of methyl groups to the homocysteine needed to regenerate SAM; (2) the assumed involvement of SAM in the metabolism of diethanolamine to methyldiethanolamine and dimethyldiethanolamine (see Figure 3). As methyldiethanolamine and dimethyldiethanolamine do not continue to participate in the endogenous transfer of methyl groups, they remove methyl from homoeostasis. This alters DNA methylation, for example, resulting in changes in gene expression, cell proliferation, apoptosis, reduced intercellular communication via gap junctions and cell transformation with subsequent tumour progression. Evidence of the methylation of diethanolamine has been found also in human liver sections and human plasma. However, the role played by the oxidation of choline to betaine varies from species to species and is a major pathway in rodents, but a minor one in humans. Humans rely more on tetrahydrofolate for maintaining SAM levels. Mice seem to be more sensitive to the effects of SAM than rats. Mouse and human hepatocytes exhibited distinct species differences after exposure to diethanolamine. Unlike in mouse hepatocytes, in human hepatocytes neither an increase in DNA synthesis, nor a decrease in intercellular communication via gap junctions were observed (Kirman et al. 2016).

Tumours may not have developed in rats under the conditions of the NTP carcinogenicity studies in part because dermal absorption is lower in rats than in mice. On the basis of the amount of dose absorbed, the systemic exposure of rats to diethanolamine was about 2 to 5 times lower than that of mice. Furthermore, B6C3F1 mice are particularly sensitive to hypomethylation stress, a factor that may contribute to their very high background incidence of liver tumours. As a result, rats may require exposure to much higher doses of diethanolamine for tumour induction than mice (Kirman et al. 2016).

Overall, the studies that investigated metabolism have shown that the liver tumours induced by diethanolamine in mice may likewise be induced by methyldiethanolamine. The liver tumours are of questionable relevance to humans because humans have metabolic pathways that differ from those of mice. Therefore, if tumours are induced in humans at all, there are distinct quantitative differences. What role the diethanolamine-induced disturbance in choline homoeostasis plays in the development of kidney tumours in mice has not been investigated. The same mechanism of action is assumed, and it cannot be ruled out at this time that carcinogenic effects induced by diethanolamine and methyldiethanolamine on the kidneys may be relevant to humans.

## 3 Toxicokinetics and Metabolism

### 3.1 Absorption, distribution, elimination

A study carried out in 1996 investigated the dermal absorption of ^14^C-methyldiethanolamine in groups of 4 male and 4 female Fischer 344 rats after a single dose of 500 mg/kg body weight. The substance was applied occlusively to the shaved skin of the interscapular area. In one group, skin contact with the applied dose was maintained for 72 hours, while in the other group the applied dose was removed after 6 hours. Samples were taken from the liver, kidneys, bone marrow, spleen, brain, heart, lungs, muscle, fat, uterus and ovaries or testes. The cages were washed, and the wash was collected. The substance was absorbed well by both male and female rats (17%–21% and 41%–50% after 6 hours and 72 hours of contact, respectively) after topical application; it appears to have been first absorbed through the skin and later slowly released into the circulating blood. The distribution of radioactivity throughout the major organs was relatively uniform, with the highest concentrations being found in the liver and kidneys. The primary route of excretion of the substance in its metabolized form was the urine with an elimination half-life of more than 30 hours (no other details; ECHA 2022). In the described tests, the substance was applied to an area of 8 cm^2^ on the males and to an area of 6 cm^2^ on the females. The amounts of undiluted methyldiethanolamine applied were calculated to be 108 and 81 mg, respectively, based on the dose of 500 mg/kg body weight and the average body weights of the males (216 g) and females (162 g). Fluxes of 2.835 mg/cm^2^ in 6 hours and of 0.473 mg/cm^2^ and hour were calculated for both male and female animals taking into account that 21% of the applied amount of substance was absorbed over a period of 6 hours.

A study carried out in 1996 investigated the kinetics and the distribution of ^14^C-methyldiethanolamine in the tissues of groups of 4 male Fischer 344 rats following treatment with a single intravenous dose of 50 or 500 mg/kg body weight. The animals were kept in metabolism cages for 72 hours, and the urine, faeces and exhaled ^14^CO_2_ were collected. The highest concentrations of ^14^C-methyldiethanolamine were found in the liver and kidneys; the distribution of the substance throughout the remaining organs was relatively uniform. The primary, but slow route of excretion of the substance was via the urine in its metabolized form. The data showed that metabolism was saturated at the high dose (no other details; ECHA 2022). The kinetics cannot be assessed because there are no data for the concentration and time.

### 3.2 Metabolism

There are no studies available for methyldiethanolamine.

Methyldiethanolamine is a metabolite of diethanolamine that forms during metabolism via *N*-methylation. Therefore, its further metabolism follows the same pathway as described for diethanolamine in Section 2.

## 4 Effects in Humans

Data are available only for sensitizing effects.

### 4.1 Sensitizing effects on the skin

In a study carried out in 5 centres of the Information Network of German Departments of Dermatology (Informationsverbund Dermatologischer Kliniken (IVDK)) between April 2000 and July 2002, a total of 229 workers in the metal industry were patch tested with methyldiethanolamine as a 1% formulation in petrolatum. Test formulations of the substance are not commercially available. The study was carried out in dermatitis patients with ongoing or past exposure to water-miscible metal-working fluids. Readings were taken at least up to day 3 (no other details). On day 3, one of the 229 tested persons (0.4%) produced a 1+ reaction (Geier et al. 2003).

#### Individual cases

A 61-year-old borer and filer machinist had dermatitis on his hands that had developed in the early 1990s, at least 6 years after he had started work. Patch tests carried out in 1992 and 2001 with the metal-working fluids used at that time yielded negative results. In further patch tests carried out in 2012 with the metal-working fluid currently being used and some ethanolamines, the patient did not react to methyldiethanolamine (tested concentration not reported), but reacted to the metal-working fluid (1+ to 1%, 3.2% and 10% test formulations). He produced a questionably positive reaction to monoethanolamine (tested concentration not reported) and reacted to other components of the metal-working fluid (Suuronen et al. 2015). The publication does not explain why the tests were carried out in 2001 and 2012, but it is assumed that they were performed because of recurrent or persisting dermatitis.

Furthermore, the case of a 38-year-old female machinist was reported who had dermatitis on her hands, forearms and face that markedly worsened upon her return from maternity leave. After initial patch test results with formaldehyde, nickel, glyoxal and several ethanolamines yielded positive results, tests were carried out with the components of the metal-working fluid that she used most frequently. She reacted to monoethanolamine (1+ reaction to 2%; vehicle not reported) and other substances (Suuronen et al. 2015).

In another case, for 5 months a CNC (computerized numerical control) machine operator had had recurrent eczema on 2 fingers of each hand at the workplace. The patch test yielded weakly positive reactions to the metal-working fluid used. Further patch tests were carried out with components of the metal-working fluid. After 3 days, the worker had not reacted to 1% methyldiethanolamine in petrolatum, but positive results were obtained with *N*-butyl-1,2-benzisothiazolin-3-one (Dahlin and Isaksson 2015).

The results of the patch tests are shown in Table 1.

**Tab. 1:** Reported test results from patch tests with methyldiethanolamine

Tested persons	Concentration, vehicle, readings	Result: reaction	Remarks	References
229 dermatitis patients with ongoing or past exposure to water-miscible metal-working fluids	1%, petrolatum, at least up to D3 (no other details)	1 × 1+ (0.4%)		Geier et al. 2003
61-year-old borer machinist with recurrent or persistent dermatitis on both hands	concentration and vehicle not reported, D2, D3 and D4 or D2, D3, D6 or D2 and D5 (unclear data)	negative	the patient reacted to the metal-working fluid; however, it was not reported whether the fluid contained methyldiethanolamine	
38-year-old female machinist with dermatitis on her hands, forearms and face	2%, no data, D2, D3 and D4 or D2, D3, D6 or D2 and D5 (unclear data)	1+	methyldiethanolamine was a component of the metal-working fluid that was used most frequently at the time	Suuronen et al. 2015
metal worker with recurrent eczema on 2 fingers of each hand	1%, petrolatum, D3 and D7	negative	methyldiethanolamine was a component of the metal-working fluid used	Dahlin and Isaksson 2015

D: day reading was taken

### 4.2 Sensitizing effects on the airways

There are no data available.

## 5 Animal Experiments and in vitro Studies

### 5.1 Acute toxicity

#### 5.1.1 Inhalation

In earlier studies carried out in the 1950s and 1960s, rats tolerated exposure to an atmosphere saturated with methyldiethanolamine for a period of 8 hours without producing any symptoms. None of the animals died. Necropsy did not yield any unusual findings. LC_50_ values were not determined (Greim 1998).

The same result was obtained in an inhalation hazard test in which groups of 5 male and 5 female Sprague Dawley rats were exposed whole-body for 6 hours to an atmosphere saturated with methyldiethanolamine. The observation period was 14 days. Mortality, clinical signs of toxicity or effects on body weights were not observed. The gross-pathological examination did not yield any unusual findings (Ballantyne and Leung 1996).

#### 5.1.2 Oral administration

In studies carried out in the 1950s and 1960s, an oral LD_50_ greater than 4000 mg/kg body weight was determined for methyldiethanolamine in rats (Greim 1998).

An LD_50_ of 1945 mg/kg body weight was obtained for methyldiethanolamine in a study carried out in groups of 5 male and 5 female Sprague Dawley rats (doses not reported). The signs of toxicity were lethargy (from 2 minutes to 2 days after administration), lacrimation (from 1.5 hours to 1 day after administration), chromodacryorrhoea (after 1 day), diarrhoea (after 30 minutes), kyphosis (from 1.5 hours to 1 day after administration) and exhaustion (after 1 day). The necropsy of the animals that died revealed distended stomachs filled with blood, blood and congestion in the intestines and lungs with dark red spots (Ballantyne and Leung 1996).

#### 5.1.3 Dermal application

In studies carried out in the 1950s, a dermal LD_50_ greater than 5000 mg/kg body weight was determined in rabbits (Greim 1998).

Methyldiethanolamine was applied occlusively to the shaved dorsal skin of male and female New Zealand White rabbits (number of animals and dose not reported) for 24 hours. The animals were observed for 14 days. Local effects were scored 1 hour after removal of the patch and after 7 and 14 days. The LD_50_ was 10 244 mg/kg body weight for males and 11 336 mg/kg body weight for females. Deaths occurred within 2 to 12 days. Signs of toxicity included lethargy, unsteady gait, emaciation and exhaustion. The surviving animals usually recovered between days 3 and 5. The animals lost body weight in week 1. Some of them recovered from this effect during week 2 (no other details). When the patch was removed, moderate to severe erythema and oedema with ecchymosis, necrosis and ulceration were observed and either persisted until the end of the observation period or changed into scaling, alopecia and scars during week 2. The necropsy of the animals that died revealed dark red spots on the lungs, dark red livers and spots on the kidneys. In the surviving animals, the incidences of these findings were considerably lower or there were no gross-pathological findings (Ballantyne and Leung 1996).

Another study carried out in 1983 in 2 male and 2 female New Zealand White rabbits resulted in an LD_50_ greater than 2000 mg/kg body weight. No deaths were observed within the 14-day observation period (no other details; ECHA 2022).

### 5.2 Subacute, subchronic and chronic toxicity

#### 5.2.1 Inhalation

There are no data available.

#### 5.2.2 Oral administration

There are no data available.

#### 5.2.3 Dermal application

The local and systemic toxicity after repeated dermal exposure to methyldiethanolamine (purity: > 99.7%) were investigated in groups of male and female Fischer 344 rats. Two short-term studies were carried out with 11-day exposure for 6 hours a day on 5 days a week (a total of 9 applications) and a subchronic study was carried out with 13-week exposure for 6 hours a day on 5 days a week (a total of 65 applications) (see Table 2) (Werley et al. 1997). The 3 studies were carried out simultaneously by the same research group and are described below.

**Tab. 2:** Studies with repeated dermal application of methyldiethanolamine in F344 rats (Werley et al. 1997)

**Study**	**Group**	**Number of animals**		**Concentration** **[% w:v]**	**Dose ** **[mg/kg body weight and day]**
		**♂**	**♀**		
11-day study 1	control^a)^	20	20		
	low dose	20	20	undiluted	260
	middle dose	20	20	undiluted	1040
	high dose	20	20	undiluted	2080
11-day study 2	control^a)^	20	20		
	low dose	20	20	10	100
	middle dose	20	20	50	500
	high dose	20	20	75	750
13-week study	control^a)^	20^b)^	20^b)^		
	low dose	10	10	10	100
	middle dose	10	10	25	250
	high dose	20^b)^	20^b)^	75	750

^a)^ the control animals were given deionized water

^b)^ ten of the 20 animals were observed after exposure

At the end of the 11-day studies, 10 animals per sex and dose group were placed into metabolism cages, the urine was collected for 18 hours, and haematological and clinico-chemical examinations were carried out. In the 13-week study, the urine was analysed after 4 and 12 weeks and the blood was examined after 5 weeks and at the end of the study. The haematological parameters included haemoglobin, erythrocytes, haematocrit, mean erythrocyte volume (MCV), mean corpuscular haemoglobin (MCH), mean corpuscular haemoglobin concentration (MCHC), total leukocyte count, differential blood count, platelets and reticulocytes. The clinico-chemical examination determined the levels of glucose, urea nitrogen, creatinine, total protein, bilirubin, calcium, sodium, potassium, chloride, phosphorus, aspartate aminotransferase (AST), alanine aminotransferase (ALT), creatine kinase, lactate dehydrogenase, γ-glutamyltransferase, sorbitol dehydrogenase and alkaline phosphatase. A protein electrophoresis test was carried out. The urine was examined for its volume, colour, microscopic components, pH, osmolality, protein, glucose, ketones, bilirubin, urobilinogen and blood. Additional parameters investigated in the subchronic study were creatinine, creatinine clearance, alpha2u-globulin, *N*-acetyl-β-D-glucosaminidase, body weights, and feed and water consumption. A complete necropsy was performed, and the liver, kidneys, brain, heart, adrenal glands, spleen, ovaries and testes were weighed. The histopathological examination included a large number of tissues (no other details) and the skin at the application site (Werley et al. 1997).

In the first 11-day study, undiluted methyldiethanolamine caused dose-dependent skin irritation (see Table 3), which presumably resulted in haematological and clinico-chemical changes (see Table 4). The body weight gains were reduced in a dose-related manner at dose levels of 260 mg/kg body weight and above; this effect reached statistical significance in the males (see Table 5), but not in the females. At the middle dose and above, the feed consumption of the males was reduced with statistical significance within the first week. In the females, the absolute and relative kidney and adrenal gland weights were increased with statistical significance in the high dose group and the relative kidney weights also in the middle dose group. The absolute and relative adrenal gland weights were increased by 12% and 16%, respectively. The absolute kidney weights were increased by 10% in the high dose group and the relative kidney weights were increased by 5% and 14%, respectively, in the middle and high dose groups. The histopathological examination was limited to the area of treated skin. Acanthosis and hyperkeratosis were observed and increased in incidence and severity in relation to the dose. In addition, multifocal areas with superficial dermatitis and exocytosis of polymorphonuclear leukocytes into the stratum corneum were found. The LOAEL (lowest observed adverse effect level) of this study was 260 mg/kg body weight and day (Werley et al. 1997).

**Tab. 3:** Skin findings after 9 dermal applications (11-day study 1) of undiluted methyldiethanolamine in F344 rats (Werley et al. 1997)

**Effects**	**Sex**	**Dose [mg/kg body weight and day]**			
		**0**	**260**	**1040**	**2080**
exfoliation	♂	0	5^a)^ (4–11)^b)^	14 (4–12)	17 (4–12)
	♀	0	19 (4–12)	20 (4–12)	20 (4–12)
excoriation	♂	0	12 (5–12)	20 (5–12)	20 (5–12)
	♀	0	14 (5–12)	20 (4–12)	17 (4–12)
fissures	♂	0	0	0	0
	♀	0	0	5 (6–12)	5 (6–12)
necrosis	♂	0	1 (5–12)	9 (6–12)	16 (5–12)
	♀	0	2 (6–12)	19 (5–12)	20 (5–12)

^a)^ incidence in 20 male and 20 female animals per group

^b)^ first to last day on which the effect was observed

**Tab. 4:** Clinico-chemical findings after 9 dermal applications (11-day study 1) of undiluted methyldiethanolamine in F344 rats (Werley et al. 1997)

**Parameter**	**Sex**	**Dose [mg/kg body weight and day]**			
		**0**	**260**	**1040**	**2080**
glucose [g/l]	♂	1.06 ± 0.07^a)^	1.05 ± 0.06	1.05 ± 0.06	1.06 ± 0.08
	♀	0.99 ± 0.09	1.06 ± 0.10	1.10 ± 0.11**	1.14 ± 0.17*
urea nitrogen [mg/l]	♂	164 ± 19.6	178 ± 27.0	180 ± 21.6	174 ± 20.1
	♀	191 ± 25.1	208 ± 25.3	235 ± 32.7*	239 ± 49.5*
sodium [mmol/l]	♂	146 ± 1.3	145 ± 0.8	146 ± 0.6	146 ± 1.1
	♀	144 ± 1.4	145 ± 1.2	146 ± 1.4**	146 ± 1.1*
chloride [mmol/l]	♂	107 ± 0.9	107 ± 0.7	108 ± 1.2**	108 ± 1.1**
	♀	106 ± 1.2	107 ± 1.1	108 ± 1.1**	109 ± 1.7*

^a)^ mean ± standard deviation

*p < 0.05;

**p < 0.01

**Tab. 5:** Body weight gains (g) in F344 rats after 9 dermal applications of undiluted (11-day study 1) and diluted (11-day study 2) methyldiethanolamine (Werley et al. 1997)

Study	Sex	Day of study	Dose [mg/kg body weight and day]			
study 1			**0**	**260**	**1040**	**2080**
	♂	1–8	11.8 ± 4.11^a)^	7.3 ± 3.73**	5.6 ± 4.81**	4.6 ± 5.07**
		1–11	16.0 ± 5.30	11.8 ± 4.42*	10.3 ± 6.05**	7.5 ± 6.86**
	♀	1–8	6.2 ± 1.96	5.4 ± 1.95	5.1 ± 3.02	4.1 ± 2.25
		1–11	8.8 ± 2.25	8.1 ± 2.24	7.2 ± 5.2	6.5 ± 3.14
study 2			**0**	**100**	**500**	**750**
	♂	1–8	12.2 ± 4.81	10.6 ± 3.09	9.8 ± 3.88	9.1 ± 6.91
		1–11	17.3 ± 5.57	16.1 ± 3.76*	15.5 ± 3.49	16.0 ± 3.98
	♀	1–8	8.7 ± 2.38	7.2 ± 1.56*	7.8 ± 2.63	6.0 ± 2.1**
		1–11	12.2 ± 3.34	11.8 ± 1.77	11.8 ± 1.77	10.3 ± 2.76

^a)^ mean ± standard deviation

* p < 0.05;

** p < 0.01

In the second 11-day study that investigated aqueous dilutions of methyldiethanolamine (10%, 50% and 75%), a slight decrease in body weight gains was observed (see Table 5) that, however, reached statistical significance only in the females of the high dose group in week 1. The body weight gains of the males were reduced in relation to the dose, but not with statistical significance. Local irritation was observed in the middle and high dose groups, resulting in statistically significant changes to clinico-chemical parameters (see Table 6). In these groups, a statistically significant increase in adrenal gland weights was observed that was likewise attributed to the local irritation. Mild erythema was found in both the males and the females. In 11 of 20 male animals, exfoliation (scaling and detachment) and excoriation (tissue defect with damage reaching into the dermis) were initially observed in the area of affected skin on day 4 or 5 after treatment with the high dose of 750 mg/kg body weight. In the females, exfoliation was found in all animals of the middle and high dose groups from day 4 of treatment. Excoriation was observed in 4 and 16 of 20 females in the middle and high dose groups, respectively, from day 6 of treatment. AST and ALT were increased with statistical significance in the blood of the males exposed to 750 mg/kg body weight and day and the sorbitol dehydrogenase and calcium concentrations were reduced with statistical significance at the middle dose of 500 mg/kg body weight and day and above. In the females of the high dose group, total protein and albumin were increased with statistical significance and sorbitol dehydrogenase and inorganic phosphorus were reduced with statistical significance (see Table 6). The relative adrenal gland weights were increased with statistical significance in the females of the middle (15.4%) and high (12.8%) dose groups. No substance-induced effects on the haematological parameters or urinalysis were found. Histopathological findings were observed only on the treated skin and consisted of acanthosis and hyperkeratosis that increased in incidence and severity in relation to the dose, multifocal dermatitis and exocytosis of polymorphonuclear leukocytes into the stratum corneum. There were no signs of systemic toxicity. The local NOAEL (no observed adverse effect level) was 100 mg/kg body weight and day (about 10% solution), and the systemic NOAEL was 750 mg/kg body weight and day. Irritation was observed 4 days after treatment with the 50% and 75% solutions (Werley et al. 1997).

**Tab. 6:** Clinico-chemical findings after 9 dermal applications of diluted methyldiethanolamine (11-day study 2) in F344 rats (Werley et al. 1997)

**Clinico-chemical parameter**	**Sex**	**Dose [mg/kg body weight and day]**			
		**0**	**100**	**500**	**750**
AST [IU/l]	♂	66 ± 4.9^a)^	69 ± 6.2	64 ± 5.7	85 ± 21.1**
	♀	69 ± 4.5	73 ± 8.8	73 ± 0.11**	76 ± 11.0
ALT [IU/l]	♂	27 ± 3.5	28 ± 4.7	25 ± 2.5	35 ± 11.7**
	♀	20 ± 3.0	23 ± 4.5	22 ± 3.5	22 ± 4.3
sorbitol dehydrogenase [IU/l]	♂	11 ± 5.7	9 ± 2.8	4 ± 1.7*	7 ± 2.5**
	♀	11 ± 5.0	16 ± 7.6**	7 ± 3.6	6 ± 3.2**
calcium [mg/l]	♂	105 ± 3.3	102 ± 3.4**	102 ± 2.2**	102 ± 3.0*
	♀	100 ± 8.2	99 ± 6.4	101 ± 4.6	104 ± 1.8
total protein [g/l]	♂	62 ± 2.5	62 ± 2.4	61 ± 3.1	62 ± 2.5
	♀	57 ± 1.5	58 ± 1.4**	57 ± 2.2	59 ± 1.9*
albumin [g/l]	♂	35 ± 1.7	35 ± 1.6	35 ± 2.0	35 ± 1.7
	♀	34 ± 1.0	34 ± 0.9	33 ± 1.0	35 ± 1.2**
inorganic phosphorus [mg/l]	♂	98 ± 3.0	95 ± 4.6	97 ± 7.4	97 ± 6.3
	♀	108 ± 4.3	109 ± 6.4	106 ± 5.1	104 ± 5.4**

^a)^ mean ± standard deviation

* p < 0.05;

** p < 0.01

The 13-week study was likewise carried out with aqueous dilutions of methyldiethanolamine (10%, 25% and 75%) in doses of 0, 100, 250 or 750 mg/kg body weight and day. The middle dose was reduced to 250 mg/kg body weight and day because of the results of the 11-day study. Ten animals of the high dose group were observed for 4 weeks. No deaths or signs of systemic toxicity were observed. There were no significant differences in the absolute body weights of either sex or in the body weight gains of the females compared with the values determined in the control animals. The changes in body weight gains observed in the males were transient, variable and generally restricted to the first 7 weeks of the study: there was a decrease in weeks 1 to 2 at 100 mg/kg body weight and day, decreases in weeks 1 to 2 and 5 to 6, followed by increases in weeks 6 to 7 at 250 mg/kg body weight and day, and decreases in weeks 1 to 2 and 4 to 5, with increases in weeks 2 to 3, 6 to 7 and 15 to 16 at 750 mg/kg body weight and day (no other details). These findings cannot be considered adverse because they were observed in only one sex and were only transient. The treated and control animals did not differ in the results of the haematological examinations, clinical chemistry, urinalysis or in the organ weights.

Exfoliation, excoriation, ulceration and necrosis were observed at 250 mg/kg body weight and day and above. The incidences and severity of these findings were dependent on the time and dose. Histopathological findings were restricted to the treated skin. Acanthosis, hyperkeratosis and parakeratosis were the most common lesions. These effects were observed in the females in the middle dose group and in both sexes in the high dose group. There was evidence of minimal to marked dermal fibrosis and dermatitis at 250 mg/kg body weight and day and above; the females were again somewhat more susceptible to these effects. At 750 mg/kg body weight and day, mild erythema was additionally observed in a few animals over a limited period of time: in the females from days 2 to 7 and in the males on days 2 and 3 and from days 68 to 70.

Overall, the only systemic effect that was observed at 100 mg/kg body weight and day was a slight decrease in body weight gains in the males during the first 2 weeks. Local effects were not evident at this dose level. Therefore, the local NOAEL was 100 mg/kg body weight and day (Werley et al. 1997). The systemic NOAEL was 750 mg/kg body weight and day.

### 5.3 Local effects on skin and mucous membranes

#### 5.3.1 Skin

In tests carried out in the 1950s to investigate skin irritation in 5 albino rabbits, 0.01 ml undiluted methyldiethanolamine induced slight irritation after non-occlusive application to the shaved skin for 24 hours. A study carried out in the 1960s did not observe reactions on the rabbit skin up to 15 minutes after the application of undiluted methyldiethanolamine. Erythema and oedema were reported after contact with the substance for 20 hours (Greim 1998).

After 4-hour occlusive application of 0.5 ml undiluted methyldiethanolamine to the shaved dorsal skin of New Zealand White rabbits, the irritation index (24, 48 and 72 hours) for erythema and oedema was 0.2 of a maximum of 4. The effects were completely reversible within 72 hours (no other details; Ballantyne and Leung 1996).

Overall, the available acute studies found that methyldiethanolamine causes mild irritation on the rabbit skin. Irritation of the skin was likewise observed in studies with repeated dermal exposure of F344 rats (see Section 5.2.3).

The substance does not cause irritation of the skin according to GHS criteria (ECHA 2022).

#### 5.3.2 Eyes

In a study carried out in the 1950s, the instillation of 0.02 ml undiluted methyldiethanolamine into the rabbit eye caused marked irritation. In a study carried out in the 1960s, redness, swelling, corneal opacity and conjunctival bleeding were observed after the instillation of 0.05 ml undiluted methyldiethanolamine into the rabbit eye. The symptoms were no longer detectable after 8 days (Greim 1998).

Amounts of 0.005 ml undiluted methyldiethanolamine were instilled into 1 eye of 6 New Zealand White rabbits. Mild to moderate redness and swelling were observed within 1 hour, but were reversible within 1 to 3 days. Mild injection of the iris was detected, which likewise persisted for about 3 days. Corneal opacity, which affected only ¼ or less of the surface area, was observed in 1 of the 6 animals 24 hours after treatment with the test substance and was reversible within 3 days. The irritation scores were 0.8, 0.3, 0.3 and 0,0 for erythema and 0.7, 0.3, 0.3 and 0.0 for oedema after 1, 24, 48 and 72 hours, respectively (no other details; Ballantyne and Leung 1996).

Overall, methyldiethanolamine caused irritation in the rabbit eye.

The substance was classified in “Eye Irritation Cat. 2, H319, causes serious eye irritation” according to GHS criteria (ECHA 2022).

### 5.4 Allergenic effects

#### 5.4.1 Sensitizing effects on the skin

A maximization test was carried out in 20 guinea pigs (Dunkin Hartley) according to OECD Test Guideline 406. Intradermal induction was carried out with 5% test substance dissolved in propylene glycol, and the undiluted test substance was used for topical induction. After provocation with 10% and 50% test concentrations (vehicle: 0.9% physiological saline or 70% ethanol; information not clear), no skin reactions were observed either in the treated animals or in the control animals. The first provocation tests with the undiluted test substance had to be repeated because of irritation (reactions in 18 of 20 animals of the treatment group and in 10 of 10 of the control group) (Leung and Blaszcak 1998). Therefore, the test results were regarded as negative.

Tests with new approach methods are not available.

#### 5.4.2 Sensitizing effects on the airways

There are no data available.

### 5.5 Reproductive and developmental toxicity

In a screening test for reproductive and developmental toxicity carried out with methyldiethanolamine (purity: > 99.9%) according to OECD Test Guideline 421, groups of 10 male and 10 female Wistar rats (Crl:WI(Han)) were given the substance as an aqueous solution in gavage doses of 0, 100, 300 or 1000 mg/kg body weight and day. The control animals received only the vehicle drinking water. Treatment began for both sexes 2 weeks before mating and continued throughout the mating period (maximum of 2 weeks). The females were treated during the entire gestation period until 4 days after parturition. The offspring were sacrificed and examined on postnatal day 4; the dams were sacrificed shortly thereafter.

At doses of 300 mg/kg body weight and day and above, the body weight gains of the male parental animals were reduced with statistical significance (weeks 0 to 3: 29% lower than those of the controls; weeks 2 to 3: 57%) and the terminal body weights were reduced in the male and female parental animals (300 mg/kg body weight and day: males 4% lower than those of the controls and females 5% lower than those of the controls; 1000 mg/kg body weight: 7% and 5%, respectively). At 1000 mg/kg body weight and day, the body weight gains of the dams were reduced during gestation (46% lower than those of the controls) and the body weights were decreased between gestation days 14 and 20 (14% lower than those of the controls). At this dose level, the duration of gestation was increased with statistical significance compared with that of the controls (22.8 days; controls: 21.9 days). In addition, at 1000 mg/kg body weight and day, litter loss was recorded for 4 dams and undelivered offspring were palpable in 2 dams. The examination revealed a decreased number of implantation sites (6.7; controls: 12.9) and increased post-implantation losses (31%; controls: 6%). The number of live offspring was decreased (4.6; controls: 12.1) and the viability index was lower (62%; controls: 99%). The reduced viability index was attributed to dead and cannibalized offspring.

At 1000 mg/kg body weight and day, the dams consumed 37% less feed during the lactation period. At the same time, a loss in body weight of 3.9 g was recorded between lactation days 0 and 4, and the body weights were about 5% lower than those of the control animals on lactation day 4. Two dams exhibited impaired lactation behaviour (little or no milk was found in the stomachs of the offspring). The body weight gains of the offspring were reduced (52% lower than those of the controls) and the body weights were reduced on postnatal day 4 (about 20% lower than those of the controls).

The NOAEL for parental toxicity was 100 mg/kg body weight and day based on the effects on body weights. The NOAEL for reproductive performance and fertility was 300 mg/kg body weight and day because of litter loss, impaired lactation behaviour and the increased duration of gestation. The NOAEL for perinatal toxicity was 300 mg/kg body weight and day based on the decreased survival index and the reduced body weight gains of the offspring. Therefore, effects on the offspring were observed only with concurrent parental toxicity (BASF SE 2010).

The findings of this study are not sufficient to draw conclusions about developmental toxicity because the study was carried out according to OECD Test Guideline 421 and this guideline does not include a full evaluation of developmental toxicity.

### 5.6 Genotoxicity

#### 5.6.1 In vitro

A mutagenicity assay used the pre-incubation method to test methyldiethanolamine (purity: 98%) concentrations of up to 10 000 µg/plate in the Salmonella typhimurium strains TA98, TA100, TA1535 and TA1537. The tests were carried out both with and without a metabolic activation system (S9 fraction from livers of male Sprague Dawley rats and Syrian hamsters treated with Aroclor 1254). There was no evidence of mutagenic properties (Greim 1998).

Another mutagenicity test in the Salmonella typhimurium strains TA98, TA100, TA1535, TA1537 and TA1538 investigated concentrations up to 10 000 µg/plate (purity: 99.8%) both with and without the addition of a metabolic activation system; cytotoxicity was observed at concentrations of 3000 µg/plate and above without metabolic activation and at concentrations of 10 000 µg/plate and above with metabolic activation. Methyldiethanolamine was not found to be mutagenic in this study (Leung and Ballantyne 1997).

In an HPRT test in CHO cells, methyldiethanolamine (purity: 99.8%) did not induce mutagenic effects either in concentrations up to 2000 µg/ml without the addition of a metabolic activation system or in concentrations up to 33 000 µg/ml with the addition of a metabolic activation system. No cytotoxicity was observed up to the highest concentration (Leung and Ballantyne 1997).

A sister chromatid exchange (SCE) assay carried out with methyldiethanolamine (purity: 99.8%) found a significant increase in the number of SCEs without the addition of a metabolic activation system in one of the duplicate tests performed with the lowest concentration tested (300 µg/ml) and in both duplicates of the middle (600 µg/ml) and high (1000 µg/ml) concentrations. However, the effects were slight and not related to the concentration, and the number of SCEs was smaller than 1.5 times the control value. Methyldiethanolamine did not induce a significant increase in SCEs in concentrations of 0, 600, 1000 or 2000 µg/ml in the presence of a metabolic activation system. The concentrations did not have any effects on the mitotic index. The authors interpreted the test results as negative (Leung and Ballantyne 1997).

**Summary**: Overall, methyldiethanolamine was not found to have genotoxic properties in vitro.

#### 5.6.2 In vivo

In a micronucleus test in peripheral erythrocytes, groups of 5 male and 5 female Swiss mice were given single intraperitoneal doses of 0, 175, 350 or 560 mg/kg body weight (about 25%, 50% and 80% of the LD_50_, respectively). Blood samples were taken 24, 30, 48 and 72 hours after treatment and at least 1000 polychromatic erythrocytes per animal were examined for micronuclei. The ratio of polychromatic to normochromatic erythrocytes was unchanged and the incidence of micronuclei was not increased at any dose level or at any time (Leung and Ballantyne 1997).

**Summary**: Methyldiethanolamine did not have genotoxic effects in the mouse micronucleus test, which was the only genotoxicity test that was carried out in vivo.

### 5.7 Carcinogenicity

There are no studies available for methyldiethanolamine.

Methyldiethanolamine is a metabolite of diethanolamine, which induces carcinogenic effects in mice.

In a dermal carcinogenicity study carried out by the NTP, diethanolamine increased the incidence of liver and kidney tumours in B6C3F1 mice after treatment with the low dose of 40 mg/kg body weight and day and above. The tumour incidence was not increased in F344 rats (Hartwig 2015 b).

## 6 Manifesto (MAK value/classification)

The critical effects are local irritation and possible carcinogenic effects in the liver and kidneys.

**Carcinogenicity. **Methyldiethanolamine is the methylated metabolite of diethanolamine. In a dermal carcinogenicity study, diethanolamine increased the incidence of liver and kidney tumours in B6C3F1 mice at the low dose of 40 mg/kg body weight and day and above. It was not possible to derive a NOAEC (no observed adverse effect concentration) for the carcinogenic effects observed after dermal application. The mechanistic studies showed that methyldiethanolamine, like diethanolamine, may disrupt choline homoeostasis, which is regarded as the cause of the liver tumours. The same mechanism has been suggested for the kidney tumours, but has not been substantiated by experimental findings. Therefore, human relevance cannot be ruled out (see Section 2).

Methyldiethanolamine, like diethanolamine, has thus been classified in Carcinogen Category 3.

**Germ cell mutagenicity. **There are no studies available for germ cell mutagenicity. Methyldiethanolamine did not cause genotoxic effects in vitro in bacteria, in HPRT or SCE tests in mammalian cells or in the only genotoxicity test that was carried out in vivo, a mouse micronucleus test. Its structure does not give reason to suspect such effects. On the basis of the available data, methyldiethanolamine has not been classified in any of the categories for germ cell mutagens.

**MAK value. **It is possible to derive a MAK value for methyldiethanolamine despite its classification in Carcinogen Category 3 because it does not induce mutagenic or clastogenic effects.

There are no studies available for effects caused by repeated inhalation or oral exposure to methyldiethanolamine. After dermal exposure of F344 rats for 13 weeks, systemic toxicity was observed only at dose levels that were much higher than those at which irritation occurred. Whereas skin irritation (exfoliation, excoriation, ulceration, necrosis, acanthosis, hyperkeratosis, parakeratosis, fibrosis and dermatitis) was observed after exposure to a 25% aqueous methyldiethanolamine solution and a dose of 250 mg/kg body weight and day, systemic toxicity did not occur up to 750 mg/kg body weight and day (75% solution).

Methyldiethanolamine caused mild irritation of the skin and irritation of the eyes of rabbits.

Compared with the structurally related ethanolamines previously evaluated by the Commission (see Table 7), methyldiethanolamine lies between diethanolamine (Hartwig 2015 b) and triethanolamine (Hartwig and MAK Commission 2019) as regards its alkalinity and the irritation it causes. A MAK value of 2 mg/m^3^ has been established for methyldiethanolamine because diethanolamine (serious eye damage; MAK value: 1 mg/m^3^) is more severely irritating than methyldiethanolamine (irritation of the eye). As this concentration is below the vapour saturation level, aerosol impaction is not expected to occur; this was observed with triethanolamine (MAK value: 1 mg/m^3^) and would enhance the effect.

The margin between the MAK value of 2 mg/m^3^ and the concentration at which carcinogenic effects may occur is sufficiently large. After dermal application of diethanolamine, cell proliferation in the liver and kidneys of the sensitive mouse was observed at a dose level of 10 mg/kg body weight and day and above (Hartwig 2015 b) (converted to about 12 mg/m^3^ assuming 60% dermal absorption in mice (Knaak et al. 1997), a respiratory minute volume of 1.4 l/min/kg body weight (scaled from the respiratory minute volume of rats of 0.8 l/min/kg body weight × 7/4), 100% absorption by inhalation and 6-hour inhalation exposure). In the 13-week inhalation study in rats, effects on the liver (increased liver weights and alkaline phosphatase levels) were observed only at a concentration of 150 mg/m^3^ and above (Hartwig 2015 a). Mice react with greater sensitivity than rats to a disturbance in choline homoeostasis, and rats in turn are considerably more sensitive than humans. As a result, there is a sufficient margin between the MAK value of 2 mg/m^3^ and a concentration that may induce carcinogenic effects.

**Tab. 7:** Comparison of the physicochemical data, MAK values and classifications of structurally related ethanolamines with those of methyldiethanolamine

**Parameter**	**2-Aminoethanol (Hartwig and MAK Commission 2018)**	**Diethanolamine (Hartwig 2015 a, b)**	**Methyldiethanolamine**	**Triethanolamine (Hartwig and MAK Commission 2019)**
CAS number	141-43-5	111-42-2	105-59-9	102-71-6
vapour pressure	0.5 hPa, no other details	0.00037 hPa at 25 °C	0.0031 hPa at 20 °C	4.8 × 10^–6^ hPa at 25 °C
boiling point	167 °C	270 °C	243.3 °C	about 320 °C
pKa	9.5	9	8.52	7.86
vapour saturation concentration (calculated from vapour pressure)	1250 mg/m^3^	1.6 mg/m^3^	15 mg/m^3^	0.03 mg/m^3^
eye irritation	corrosive / serious eye damage	serious eye damage	irritation of the eye	not irritating
MAK value	0.2 ml/m^3^ (0.51 mg/m^3^)	1 mg/m^3^ (0.23 ml/m^3^)	2 mg/m^3^ (0.4 ml/m^3^)	1 mg/m^3^ (inhalable fraction)
peak limitation category and excursion factor	I, 1	I, 1	I, 1	I, 1
carcinogenicity	–	Category 3	Category 3	–

**Peak limitation. **Local irritation is the critical effect of methyldiethanolamine. The substance has thus been assigned to Peak Limitation Category I. As no human data are available for sensory irritation caused by methyldiethanolamine, an excursion factor of 1 has been established in analogy to that set for the other ethanolamines.

**Prenatal toxicity. **Studies of prenatal developmental toxicity are not available. In a screening test for reproductive and developmental toxicity carried out according to OECD Test Guideline 421 in Wistar rats given oral doses of methyldiethanolamine, litter loss, a reduced survival index and reduced body weight gains in the offspring were observed at 1000 mg/kg body weight and day. At the same time, maternal toxicity in the form of reduced body weight gains and decreased body weights was found. The NOAEL for perinatal toxicity was 300 mg/kg body weight and day and the NOAEL for parental toxicity was 100 mg/kg body weight and day (BASF SE 2010). The following toxicokinetic data are taken into consideration for the extrapolation of the NOAEL for perinatal toxicity to a concentration in workplace air: the daily exposure of the animals in comparison with the 5 days per week exposure at the workplace (7:5), the toxicokinetic species-specific correction value for the rat (1:4), the assumed oral absorption (100%), the body weight (70 kg) and the respiratory volume (10 m^3^) of the person, and the assumed 100% absorption by inhalation. The concentration calculated from this is 735 mg/m^3^; this is 368 times as high as the MAK value of 2 mg/m^3^. The findings of this study are not sufficient to draw conclusions about developmental toxicity because the study was carried out according to OECD Test Guideline 421 and this guideline does not include a full evaluation of developmental toxicity. Therefore, methyldiethanolamine has been classified in Pregnancy Risk Group D.

**Absorption through the skin. **The dermal absorption of methyldiethanolamine can be determined from the findings of animal studies that applied undiluted substance to the skin of rats. A flux of 0.473 mg/cm^2^ and hour can be calculated from the data. Under standard conditions (surface area of 2000 cm^2^ of skin and exposure for 1 hour), this results in an absorbed amount of 946 mg methyldiethanolamine.

The local irritation caused by the substance is decisive for the derivation of a MAK value. As a result, the systemic NOAEL of 750 mg/kg body weight and day determined from a dermal 13-week study in rats is used to estimate the potential systemic toxicity after absorption through the skin. A systemically tolerable uptake of 690 mg methyldiethanolamine was calculated for a person weighing 70 kg after taking adjustment factors into account for the extrapolation from subchronic to chronic exposure (1:2), the species-specific correction value for the rat (1:4), extrapolation from animals to humans (1:2) and the dermal absorption of 21% for rats. The amount absorbed through the skin under standard conditions thus exceeds the tolerable uptake. Therefore, methyldiethanolamine has been designated with an “H” (for substances which can be absorbed through the skin in toxicologically relevant amounts).

**Sensitization. **Only few findings are available for the skin sensitizing potential of methyldiethanolamine. The findings suggest that methyldiethanolamine does not cause pronounced contact sensitization. A maximization test with a 50% test formulation yielded negative results. Tests using new approach methods are not available. There are no data available for sensitizing effects on the respiratory tract. Methyldiethanolamine has therefore not been designated with either “Sh” or “Sa” (for substances which cause sensitization of the skin or airways).
